# Optimizing human Treg immunotherapy by Treg subset selection and E-selectin ligand expression

**DOI:** 10.1038/s41598-017-17981-z

**Published:** 2018-01-11

**Authors:** Conor Donnelly, Brad Dykstra, Nandini Mondal, Junning Huang, Belinda J. Kaskow, Russell Griffin, Robert Sackstein, Clare Baecher-Allan

**Affiliations:** 1 Ann Romney Center for Neurologic Disease, Harvard Medical School, Boston, MA 02115 USA; 2 Department of Dermatology, Brigham and Women’s Hospital, Harvard Medical School, Boston, MA 02115 USA; 3 Department of Medicine, Brigham and Women’s Hospital, Harvard Medical School, Boston, MA 02115 USA; 4 Program of Excellence in Glycosciences, Harvard Medical School, Boston, MA 02115 USA; 5 University of Pittsburgh School of Medicine, Pittsburgh, PA USA; 6 Platelet Biogenesis, Boston, MA USA

## Abstract

While human Tregs hold immense promise for immunotherapy, their biologic variability poses challenges for clinical use. Here, we examined clinically-relevant activities of defined subsets of freshly-isolated and culture-expanded human PBMC-derived Tregs. Unlike highly suppressive but plastic memory Tregs (memTreg), naïve Tregs (nvTreg) exhibited the greatest proliferation, suppressive capacity after stimulation, and Treg lineage fidelity. Yet, unlike memTregs, nvTregs lack Fucosyltransferase VII and display low sLe^X^ expression, with concomitant poor homing capacity. *In vitro* nvTreg expansion augmented their suppressive function, but did not alter the nvTreg sLe^X-l^^°^^w^ glycome. However, exofucosylation of the nvTreg surface yielded high sLe^X^ expression, promoting endothelial adhesion and enhanced inhibition of xenogeneic aGVHD. These data indicate that the immature Treg glycome is under unique regulation and that adult PBMCs can be an ideal source of autologous-derived therapeutic Tregs, provided that subset selection and glycan engineering are engaged to optimize both their immunomodulation and tropism for inflammatory sites.

## Introduction

The ability to achieve immunomodulation via cellular therapy offers the promise of avoiding pharmacologic immunosuppressants, improving the specificity of therapy and preventing toxic drug effects. Tregs represent a specialized subset of CD4^+^ T cells which co-express high levels of CD25 (interleukin-2Rα chain) and intracellular FoxP3^1^, are dependent on interleukin-2 for survival^2^, and exert dominant tolerance via a cell contact-mediated mechanism of suppression^3^. Yet, it is important to note that Treg cells obtained from patients with various autoimmune diseases have shown functional plasticity by transitioning into a pro-inflammatory state in which they secrete IFNγ or IL-17^4^. Early preclinical studies using the Treg subset of CD4^+^ T cells in immunotherapy provided evidence of their ability to dampen autoimmunity and enhance tolerance to tissue transplants^5,6^. Models of hematopoietic stem cell transplantation (HSCT) have shown that the adoptive transfer of Tregs could reduce the incidence of acute graft-versus-host disease (aGVHD)^7^ while retaining the crucial graft-versus-tumor benefit of the allotransplant, in sharp contrast to treatments with broad immunosuppressants^8^. However, as Tregs are a low frequency population and the yield of all memory Tregs is not sufficient for clinical application^9–11^, an initial *in vitro* expansion is often utilized to generate Treg cellular therapeutics.

Currently, there are over 280 clinical trials utilizing the adoptive transfer of Tregs to reduce the morbidity related to autoimmune disease, solid organ transplantation, and allogeneic hematopoietic stem cell transplantation (clinicaltrials.gov, Nov. 13, 2017). While adoptive Treg therapy represents a promising cellular therapeutic in principle, many clinical trials administering Treg populations to treat aGVHD or other immunologic diseases have not shown the efficacy that many believe Treg therapies could deliver^12^. Variations in Treg isolation procedures and expansion methods, and resulting fluctuations in Treg purities, may underlie the observed lack of efficacy. Since Tregs are a low frequency population and clinical cellular therapeutic trials require large numbers of Tregs, the trials have used Tregs that have been either enriched from large-scale leukapheresis collections or have been expanded with a period of *in vitro* stimulation. A recent report of administering autologous, *in vitro*-expanded total CD25 + CD127^10^ Tregs to recent onset T1D patients demonstrated safety but little efficacy^13^, while eight of twenty patients with Crohn’s disease showed improvement after being given autologous *in vitro*-ovalbumin-expanded clonal Tr1-like Tregs and ingesting ovalbumin to theoretically activate intestinal Tregs *in vivo* ^14,15^. Reported attempts at Treg therapy to treat aGVHD have used freshly harvested leukapheresis-derived cells in which only ~50% of the cells expressed Foxp3^10,16^, *ex-vivo* expanded cells from adult peripheral blood^11^ or third party cord-blood^9^. More recent studies have shown that delivering a Treg cellular therapeutic derived from cord blood and expanded with CD64- and CD86- expressing K562 cells reduced the number of patients exhibiting grade II-IV aGVHD by 36%^17^, and that delivering a dose of FoxP3 + Tregs before HSCT can result in an increase in disease-free survival, with the caveat of higher treatment-related mortality^18^. Although these ongoing studies hold much promise, the limitations in treatment efficacy may reflect the lack of uniformity in Treg preparation with an unclear functionality of the Treg preparations.

Identifying the ideal source of Tregs is critical to optimizing the efficacy of Treg cellular therapy^19^. An optimal Treg source or subpopulation should exhibit considerable *in vitro* expansion capacity while maintaining high purity (characterized by FoxP3 expression, not simply CD25 expression), strong Treg lineage fidelity and effective suppressive activity. Identifying the optimal starting Treg population could minimize the outgrowth of activated, non-regulatory and potentially pro-inflammatory T cells during the process of *in vitro* (and then subsequent *in vivo*) expansion. Though magnetic bead-based methods of Treg isolation are commonly used, FACS-based human Treg selection is preferable as it not only maximizes purity, but also makes it possible to isolate Treg subsets that may differ in qualities that are, or are not, desirable in a therapeutic. Comparing the functional attributes of individual Treg subpopulations may indicate if certain subsets are prone to co-express pro-inflammatory cytokines and FoxP3, while other Treg populations may be predisposed to lose FoxP3 and give rise to ‘ex-Tregs” that express self-antigen specific TCR’s but lack suppressive activity.

Three functionally-distinct Treg subsets can be FACS-sorted by virtue of CD45RA, CD25^hi^, CD127 and HLA-DR expression^20–22^: (1) The HLA-DR^+^ effector memory Tregs (DR + mTreg)^22,23^ that induce immediate strong suppression but have low proliferative capacity and short telomeres^24^; (2) The less suppressive but more proliferative HLA-DR^−^ memory Tregs (DR^−^mTreg); and (3) The least suppressive, but highly proliferative naïve Tregs (nvTreg), which become more suppressive with activation^22^. The characterization of Treg subsets for their capacity to function as an adoptive therapeutic, including proliferation with maintenance of lineage fidelity, endothelial rolling under shear forces, and capacity to undergo tissue extravasation has not been adequately described.

While it is evident that a Treg therapeutic should exhibit high purity and suppressive capacity, the ability of the infused cells to migrate into target tissues is a less obvious but similarly vital feature that can affect therapeutic efficacy. In fact, suboptimal tissue homing has been proposed to be a potential contributor to the poor efficacy reported in some Treg clinical trials^25,26^. The ability of lymphocytes to migrate into inflammatory sites is critically dependent on expression of a tetrasaccharide glycan motif known as sialyl-Lewis X tetra-saccharide (sLe^X^; CD15 s: NeuAc-α(2,3)-Gal-β(1,4)-[Fuc-α(1,3)]-GlcNac-R), which is displayed on glycoproteins and glycolipids on the lymphocyte surface. sLe^X^ is the proto-typical binding determinant for E-selectin (CD62E), an endothelial molecule. sLe^X^ can be detected by monoclonal antibodies such as CSLEX-1^27^, or by the HECA452 monoclonal which detects a determinant often referred to as “cutaneous lymphocyte antigen” (CLA). CSLEX-1 is more specific for the tetrasaccharide sLe^X^, whereas HECA452 more broadly recognizes sLe^X^ and its isomer sLe^A^, as well as sulfated, fucosylated type 2 lactosamines that can also function as E-selectin ligands^28,29^. Importantly, HECA452 staining tracks E-selectin binding activity of cells more closely than does CSLEX-1 staining^30^.

E-selectin is constitutively expressed in skin and bone marrow microvessels, and is highly expressed in microvascular endothelial beds at all sites of inflammation via its induction by inflammatory cytokines TNF-α and IL-1β^31–34^. The interaction between lymphocyte sLe^X^ and endothelial E-selectin initiates a cascade of events culminating in cell extravasation and tissue infiltration^35^. A previous study reported that freshly-isolated CD45RO+ memory Tregs stain with HECA452 (i.e. express CLA), bind to TNF-stimulated human endothelial cells and undergo transendothelial migration when tested under static conditions; in contrast, freshly-isolated CD45RA+ nvTregs were found to express little CLA and lacked the capacity to undergo static transendothelial migration^36^. Although these data suggest an association between effector state and migratory capacity, prior studies have not evaluated how *in vitro* activation of the HLA-DR and CD45RA defined Treg subpopulations affects their expression of sLe^X^ and their ability to enter tissues.

In this report, we examined the suitability of different human Treg subsets to function as a potential cellular therapeutic. We undertook a direct comparison of the *in vitro* and *in vivo* functional activities of the different memory and naïve Treg subpopulations. Both freshly-isolated and culture-expanded human peripheral blood-derived Treg populations were tested for their capacity to suppress, to engage endothelium under hemodynamic shear conditions, to extravasate through endothelium, to produce pro-inflammatory cytokines, and to proliferate with lineage fidelity. Our results indicate that the nvTreg is the Treg population that exhibits the ideal biological features of a Treg therapeutic. We also obtained data indicating that the recently described sLe^X-hi^ effector Tregs^37^ show overlapping features with effector DR + mTregs. In addition, the results indicate that the highly suppressive memTregs should be purposefully excluded from a Treg therapeutic due to their low lineage fidelity, low proliferative capacity, and greater pro-inflammatory potential. Although the nvTregs natively lack sLe^X^ expression, they display the sialylated lactosamine scaffolds that can be enzymatically modified by exofucosylation to express the sLe^X^ structure, thereby programming E-selectin binding and *in vivo* migration to inflammatory sites. Enforced expression of sLe^X^-on *in vitro* expanded nvTregs markedly enhanced the ability of the cells to inhibit *in vivo* xenogeneic aGVHD responses. Our results offer novel perspectives on the immunobiology of Treg subsets and their capacity to localize at inflammatory sites, providing a roadmap for the future selection and use of Tregs in cellular immunotherapies.

## Results

### Heterogeneity in suppressive capacity of freshly isolated human Treg subsets

**Figure 1:**
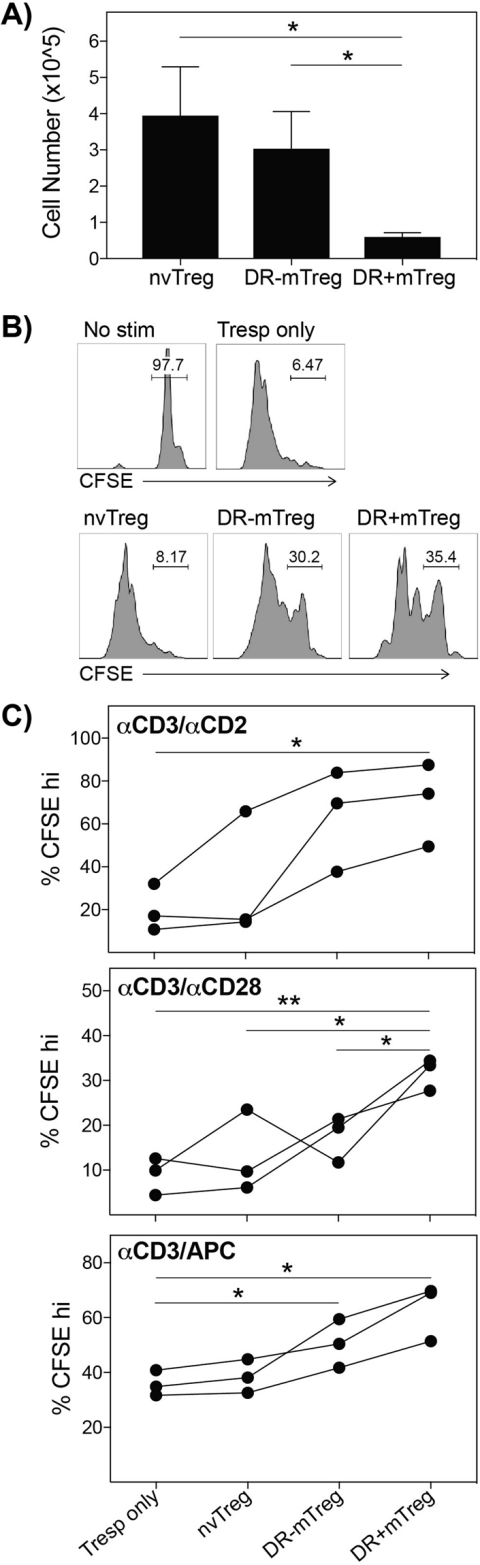
Treg subsets exhibit distinct yields and *ex vivo* suppressive capacities. (**A**) nvTregs, DR^−^mTregs, and DR^+^mTregs differ in yield when FACS-sorted from fresh donor blood (n = 7). (**B**) Typical suppression by CFSE dilution, is shown for co-cultures established with CFSE labeled Tresp and half as many Tregs (5 days). (**C**) Treg co-cultures were established under different stimulation protocols in which the cells were stimulated with αCD3/αCD2 (top), αCD3/αCD28 (middle) or anti-CD3/APC (bottom). The DR^+^mTregs induced the highest suppression, followed by DR^−^mTreg and lastly by the least suppressive nvTregs. Data shown are mean ± SEM, and significance determined by Student’s t test, is indicated as (*P < 0.05) and (**P < 0.01). [turn scatter plots into bar graphs with X+/− SEM].

As the most basic requirement for a Treg cellular therapeutic is its ability to inhibit immune activation, we directly compared the suppressive ability of FACS-sorted nvTregs, DR + mTregs and DR^−^mTregs (for FACS-gating see Supplemental Fig. 1). FACS-sorting these functionally distinct Treg populations from ~100 ml of peripheral blood from healthy donors resulted in obtaining highly pure populations (based on FoxP3 staining) exhibiting marked differences in average yield (Fig. 1A). A typical collection of 250 × 10^6^ PBMC yields 4 × 10^5^ nvTregs, 3 × 10^5^ DR^−^ mTregs and 0.5 × 10^5^ DR^+^mTregs. To compare suppressive capacity, the sorted Treg populations were co-cultured with CFSE-labeled CD25−/CD45RA− CD4^+^ T cells (mTresp) at a suboptimal, ½:1 ratio. The typical inhibition of mTresp proliferation/CFSE dilution that occurs when the mTresp cells are co-cultured with half as many of each type of Treg is shown in Fig. 1B. The sorted Treg-subsets consistently exhibited differences in suppressive ability (Fig. 1C) as the DR^+^mTregs were the most suppressive and the nvTregs exhibited the lowest suppressive capacity in co-cultures stimulated with αCD3/αCD2 (top), αCD3/αCD28 (middle), or αCD3/APCs (bottom). The finding that *ex vivo* nvTregs are less suppressive than memory Tregs is consistent with previous publications that tested the activity of suboptimal Treg numbers^38–40^, but differs from reports that assayed Treg:Tresp co-cultures at the optimal 1:1 ratio which usually results in memory and naïve Tregs inducing equal levels of suppression^41,42^.

### Treg subsets exhibit significant heterogeneity in binding to vascular endothelium under hemodynamic shear conditions

We next compared the ability of the different types of Tregs to bind and cross the endothelium as an indication of their potential capacity to migrate into target tissues *in vivo*. The process by which a T cell enters a target organ is initiated by the interaction of sLe^X^ moieties on the T cell surface with endothelial cell-expressed E-selectin, and culminates in cell extravasation and tissue infiltration^35^. Thus, we assessed whether the Treg subsets differed in their expression of surface sLe^X^, their ability to tether and roll on TNFα-stimulated vascular endothelium under fluid conditions that mimic the hemodynamic shear stress of blood flow, and to undergo static transmigration through an endothelial monolayer.

**Figure 2:**
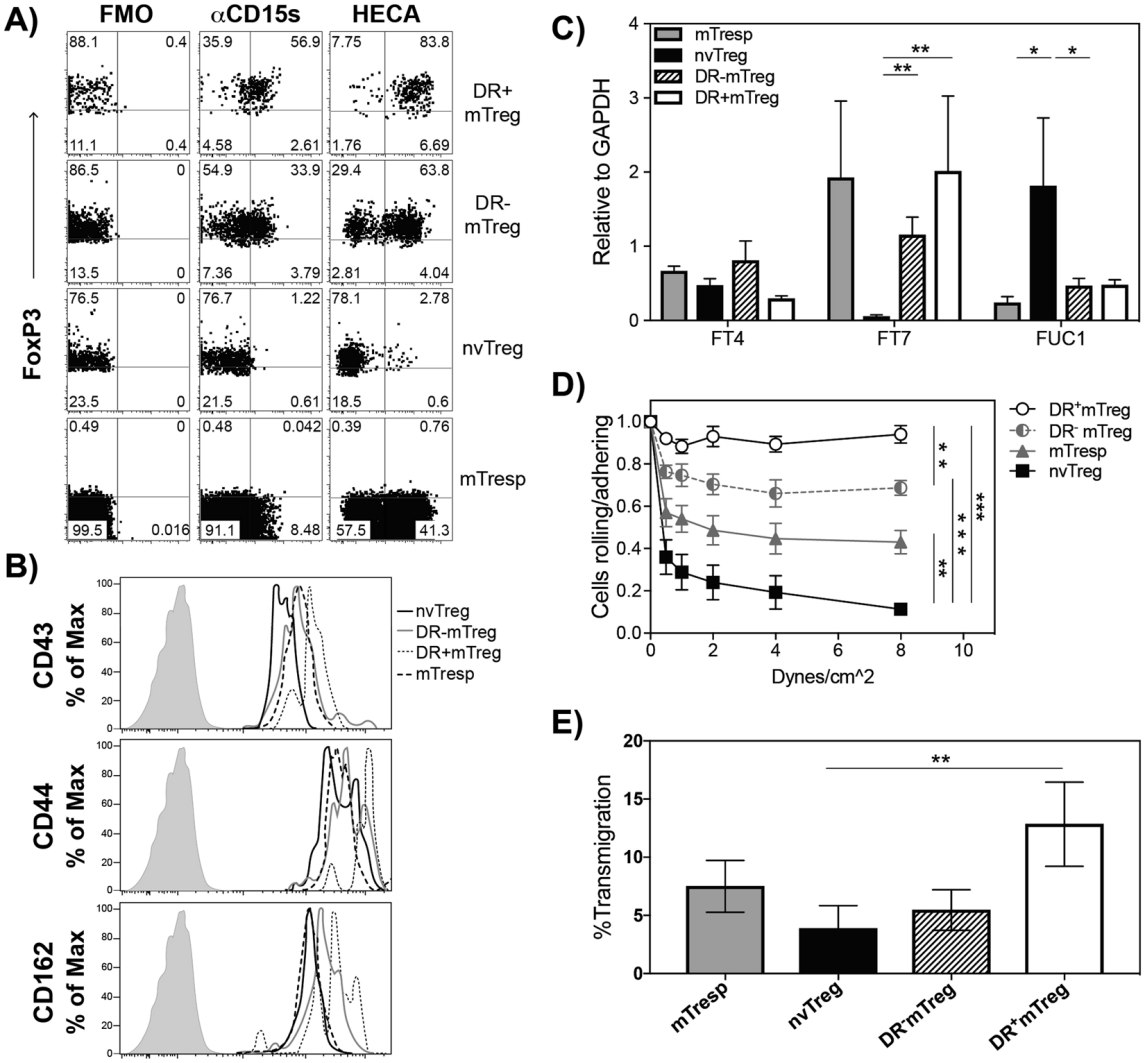
DR + mTregs and nvTregs differ in their capacity to express sLe^x^, to adhere to endothelium under shear, and to undergo endothelial transmigration. Freshly isolated cells were stained with (**A**) CD15s or HECA452 (for surface sLe^x^), or FMO control, and intracellular anti-FoxP3; or (**B**) surface expression of CD43, CD44 and PSGL1 (CD162). FACs sorted fresh cells were examined for their (**C**) relative gene expression of fucosyltransferases IV and VII and fucosidase 1α (FUC1) by RT-PCR; (**D**) capacity to roll and adhere to activated endothelium in the presence of increasing shear stress from 0.5–8 dynes/cm^2^, and their ability to (**E**) migrate through a TNFα-activated monolayer endothelial towards a CXCR4 gradient using Boyden chambers with SDF-1 (125 ng/ml) in the lower chamber (12 hours). Rolling and extravasation experiments were repeated with cells from three separate healthy donors. Data are shown as mean ± SEM. Significance was determined by Student’s t test and indicated as (*P < 0.05), (**P < 0.01), and (***P < 0.001).

First, we determined the capacity of each population to co-express intracellular FoxP3 and surface sLe^X^ as this would likely predict the ability of a cell to traffic to sites of inflammation. As sLe^X^ is detected more specifically by mAb CSLEX-1 (anti-CD15 s), but mAB HECA-452 staining has better correlation with E-selecting binding capacity^30^, both CD15 s and HECA452 antibodies were used to probe the different freshly-isolated Treg populations. Shown in Fig. 2A, the freshly-isolated blood-derived Treg subsets exhibited different patterns of sLe^X^/E-selectin-ligand expression, with CD15 s and HECA452 exhibiting highly similar binding patterns on each population. The staining with CD15 s or HECA452 mAbs indicated that the nvTregs exhibited the lowest expression of sLe^X^ (~1% CD15 s and 3% HECA452 staining), followed by the DR^−^mTregs (34% CD15 s versus 64% HECA452 staining), while the vast majority of the DR^+^mTregs strongly expressed sLe^X^ (~57% CD15 s and 84% HECA452 staining). Surprisingly, the mTresp cells expressed significantly less sLe^X^ than either of the freshly isolated memory Treg populations. Furthermore, as CD162 (PSGL1), CD44, and CD43 represent the major T-cell proteins that can be decorated with sLe^X^, the different Treg populations were also examined to determine if they exhibited similar levels of these proteins themselves. The relative staining pattern (Fig. 2B) indicated that although the DR + mTregs expressed the highest levels of each of these proteins and the highest level of CD15 s/HECA452, the nvTregs also highly expressed these protein scaffolds, but apparently, without the sLe^X^ glycosylation.

As sLe^X^ is generated via an α(1,3) linkage-specific addition of fucose to a sialo-lactosamine trisaccharide scaffold, we next examined whether the differential Treg expression of sLe^X^ reflected differences in expression of the specific fucosyltransferases that add this specific fucose linkage, or in the expression of fucosidases that can cleave fucose from the cell surface. As shown in Fig. 2C, mRNA from the different freshly isolated T cell populations was examined for relative expression of α(1,3)-fucosyltransferases IV (FUT4) and VII (FUT7), along with fucosidase 1 (FUC1). The results indicated that, although the different subsets exhibited no difference in FUT4 expression, FUT7 expression was significantly upregulated in both the DR^+^mTregs and the DR^−^mTregs relative to the nvTregs. Moreover, the nvTregs were also found to express significantly more FUC1. This combination of low fucosyltransferase activity and high fucosidase activity in the nvTreg would promote the observed low sLe^X^ expression on these cells.

The functional capacity of these freshly isolated T cell populations to roll/adhere on TNF-α−stimulated HUVEC was tested on a parallel plate flow chamber apparatus that mimics the shear forces in the circulation (Fig. 2D). Under physiologically relevant shear (1–4 dynes/cm^2^), the nvTregs exhibited the lowest capacity to bind to E-selectin^+^ endothelial cells, while increasing adherence was exhibited by the memory CD4^+^ T cells (mTresp), followed by the DR^−^ mTregs, with the highest binding activity by the DR^+^mTregs. The adhesive property of each T cell population was directly proportional to their relative expression of sLe^X^. Remarkably, the highly suppressive, DR^+^mTregs exhibited the strongest binding of any tested cell type (including tumor cell lines, data not shown), as they resisted shear up to 8 dynes/cm^2^.

Lastly, the capacity of freshly isolated Treg populations to undergo the full process of static transendothelial migration (TEM) was tested using Boyden chambers established with monolayers of HUVEC that had been stimulated with TNFα to induce E-selectin expression. As indicated in Fig. 2E, the DR^+^mTregs exhibited the most efficient transmigration through HUVEC monolayers as compared to all other freshly-isolated subsets. Unexpectedly, although the sLe^X-l^^°^^w^ nvTregs had the lowest TEM potential in which only ~3.8% underwent transmigration, the DR^−^ mTregs that are 50% positive for sLe^X^ also exhibited a surprisingly low TEM ability. Altogether, these data suggest that the DR^+^mTregs, which had previously been referred to as effector Tregs due to their rapid suppressive activity, short telomeres, and inability to proliferate^22,43^, are synonymous with the sLe^Xhi^ effector Tregs that were more recently identified by Miyara *et al*.^37^. This migratory activity indicates that the maximally suppressive DR + mTregs have the highest potential to arrive at sites of inflammation to inhibit immune activation, while the other Treg subsets would be much less likely to enter the tissue. These findings further support the effector classification of DR + mTregs as they express the highest level of sLeX and exhibit the strongest rolling, adherence, and transmigration through endothelium.

### Treg populations exhibit distinct capacities to proliferate and produce pro-inflammatory cytokines

We next examined whether the different Treg subsets exhibit distinct proliferation-associated features that would affect their suitability for selection as a cellular therapeutic. Ideally the Treg subset must be able to undergo marked *in vitro* expansion while maintaining high fidelity Treg function. Although their superior migratory activity and suppressive capacity suggest that the *ex vivo* DR^+^mTreg is the ideal population to harness for use in adoptive therapy, DR + mTregs are a rare population as they represent a minor portion (~10%) of the small circulating Treg population. In addition, DR + mTregs were previously shown to have extremely short telomeres and lack the capacity to proliferate^22^, indicating that they may not have the capacity to undergo sufficient expansion for therapeutic consideration.

**Figure 3:**
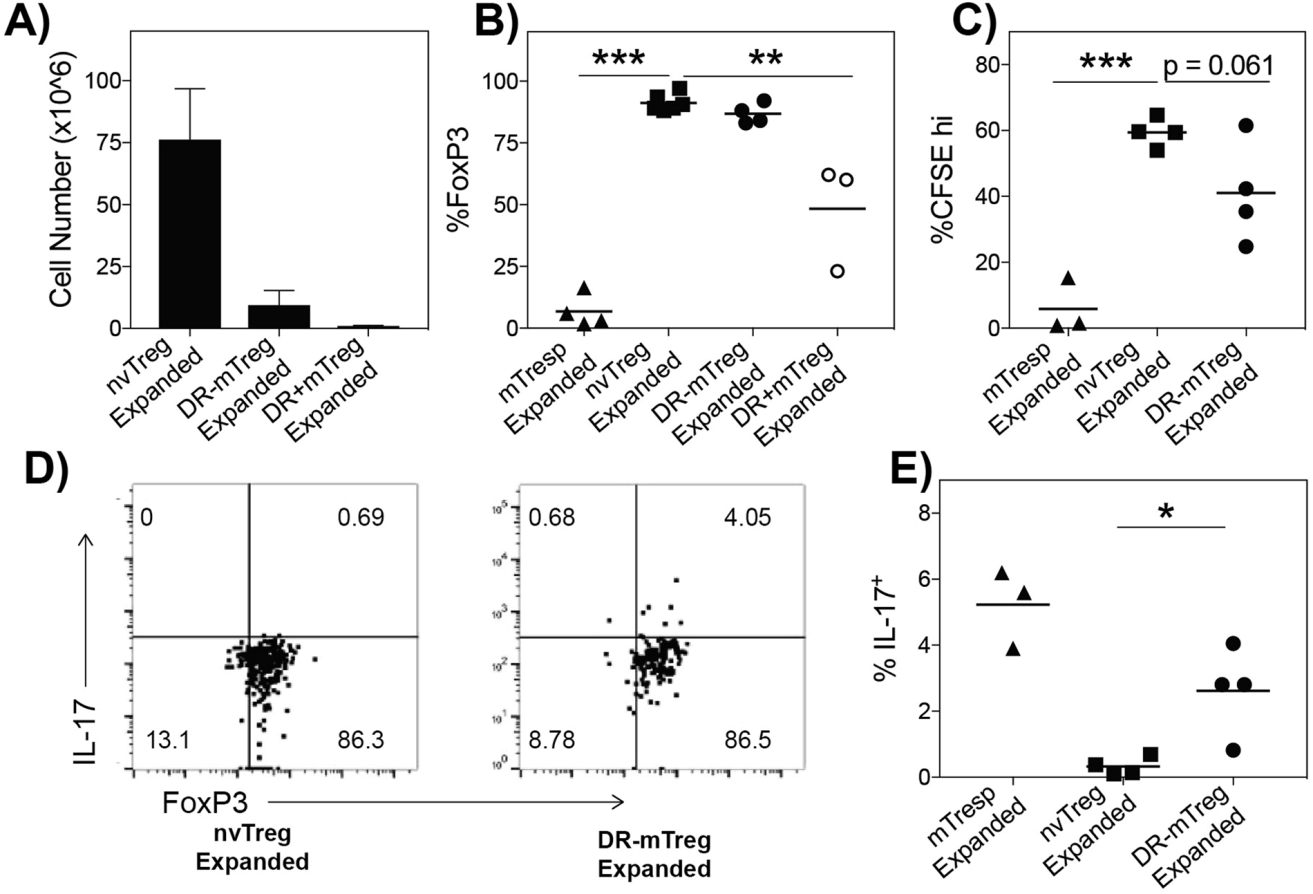
The different Treg subsets exhibit unique capacities to undergo *in vitro* expansion with maintained FoxP3 expression and suppressive activity. (**A**) The number of T cells that were produced after a 14d *in vitro* stimulation is shown (n = 3). The expanded T cell populations were tested for (**B**) expression of intracellular FoxP3 to indicate purity; and (**C**) for their ability to suppress CFSE labeled mTresp when co-cultured at a 1:1/2 (mTresp:Treg) ratio with αCD3/APC stimulation. (**D**) Shows representative intracellular staining of the expanded Tregs for co-expression of FoxP3 and IL-17. (**E**) The frequency of IL-17 producing, FoxP3+ cells in the DR^−^mTreg versus the nvTreg populations (n = 4) is shown. Data shown are mean ± SEM. Significance, determined by Student’s t test, is indicated by (*P < 0.05), (**P < 0.01), and (***P < 0.001).

In assessing the proliferative response of each FACS-sorted Treg population, the purity and lineage fidelity of each Treg population was measured after *in vitro* culture/expansion by staining for high expression of FoxP3, evaluating functional *in vitro* suppression, and testing whether the expanded cells express pro-inflammatory cytokines. A reduction in the frequency of cells expressing FoxP3^hi^ in the expanded populations would indicate either low fidelity to the Treg lineage (generation of ex-Tregs) or the expansion of contaminating non-Tregs. By counting and staining cells from each stimulated population, the nvTregs exhibited significantly higher expansion potential than either memory subset, while the DR^+^mTregs exhibited almost no growth, as expected (Fig. 3A). On average, after fourteen days of culture, the stimulation resulted in expansions of more than 200-fold (75 × 10^6^) for the nvTregs, 30-fold (10 × 10^6^) for the DR^−^mTregs, and less than 10-fold for the DR^+^mTregs. Furthermore, while the nvTreg and DR^−^mTreg consistently retained their high levels of purity, the DR^+^mTreg exhibited a surprisingly high loss of FoxP3 as just over 50% of the expanded DR + mTregs expressed FoxP3 (Fig. 3B). Thus although greater than 95% of the DR + mTregs initially expressed FoxP3, these data suggest that the cells in the stimulated DR + mTreg cultures arise from the outgrowth of non-regulatory contaminants or that these cells transition to ex-Tregs and lose FoxP3. Next, we compared the suppressive capacity of those expanded Treg populations that exhibited high lineage fidelity, the nvTregs and DR^−^mTregs. Strikingly, as shown in Fig. 3C, in contrast to the low suppressive activity of the freshly isolated blood nvTregs, the expanded nvTregs exhibited a markedly enhanced suppressive capacity that was even more profound than that exhibited by the expanded DR^−^mTregs.

As a small fraction of human Tregs have been shown to have the capacity to transition into potentially pro-inflammatory FoxP3 + cells that express either IFNγ or IL-17, the expanded high-fidelity DR^−^mTreg and nvTreg populations were tested to determine if they differed in their propensity to produce these pro-inflammatory cytokines. The ability to produce pro-inflammatory cytokines was reported to be a property of memory Tregs isolated from patients with autoimmune diseases, and has been suggested to potentially be an early indicator of decreasing lineage fidelity^44,45^. As shown in Fig. 3D (with replicates shown in Fig. 3E), the expanded DR^−^mTregs contained a low but consistent number of cells that co-expressed both IL-17 and FoxP3, while the expanded nvTregs did not co-express FoxP3 and IL-17 (IFNγ was not detected). Altogether, these data suggest the nvTregs exhibit greater fidelity to the suppressive Treg lineage than the DR^−^mTreg population.

### Exofucosylation is required to create selectin ligands on expanded nvTregs

**Figure 4:**
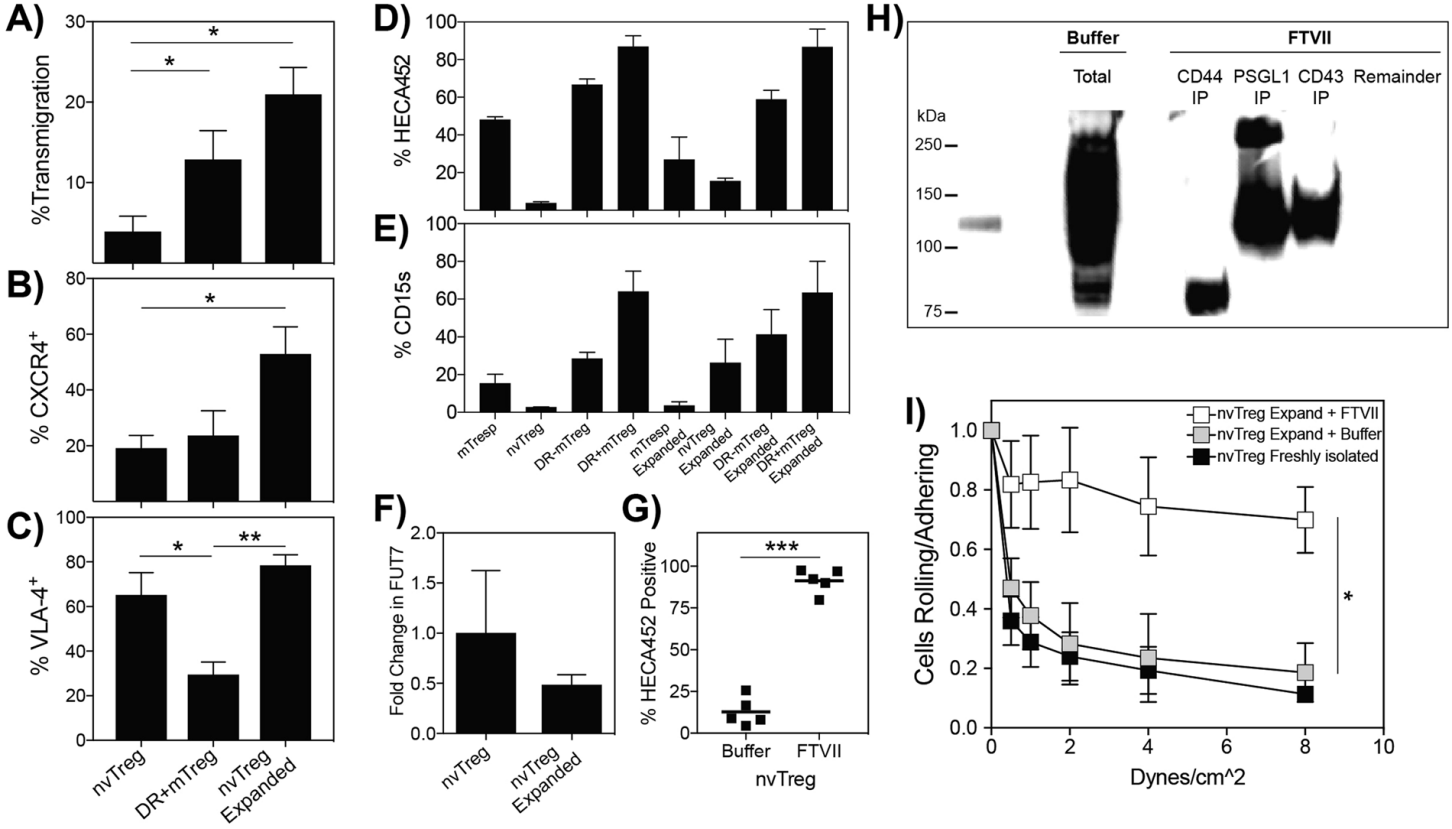
Exofucosylation increases expression of selectin ligands on nvTregs and their endothelial adherence. Freshly isolated nvTregs and effector DR^+^mTregs, and 14-day *in vitro* expanded nvTregs were tested for their ability to (**A**) undergo static transendothelial migration (TEM) towards SDF-1 in a Boyden Chamber assay, and to express surface (**B**) CXCR4, and (**C**) VLA-4. All T cell subsets, isolated from three donors, were stained pre- and post-expansion to determine expression of sLe^X^ as detected by (**D**) HECA452 or (**E**) CD15 s. (**F**) Pre- and post- *ex vivo* expanded nvTregs were examined for their relative expression of FUT7 RNA by RT-PCR. (**G**) Buffer- or fucosyltransferase VII-treated expanded nvTregs were stained to determine HECA452/sLe^x^ expression. (**H**) The proteins that bind mE-selectin-Ig in buffer- or FTVII-treated expanded nvTregs were identified by performing sequential immunoprecipitation on lysates with anti-CD44, anti-PSGL-1, and then anti-CD43, and detecting the proteins that bound mE-selectin-Ig by western blotting. The blot in Fig. 4H was not cropped as the samples were run in the order as shown. (**I**) Buffer-treated and FTVII-treated expanded nvTregs, and freshly isolated nvTregs were examined for their ability to roll and adhere to endothelium in the presence of dynamic shear forces. Data shown are mean ± SEM. Significance, determined by Student’s t test, is indicated by (*P < 0.05), (**P < 0.01), and (***P < 0.001).

As stimulated nvTregs exhibit the greatest fold expansion and highest yield, superior suppressive activity and maintained high Treg fidelity as compared to the other Treg subsets, we next asked whether the *in vitro*-expanded nvTregs would also exhibit an increased ability to undergo TEM in the absence of shear. Thus, we first tested whether the expanded nvTreg population underwent TEM in the same static migration assay described previously. As shown in Fig. 4A, a significantly higher frequency of expanded nvTregs underwent TEM as compared to the freshly isolated nvTregs. Surprisingly, the TEM capacity exhibited by the expanded nvTregs was even greater than the TEM capacity exhibited by the freshly-isolated “effector” DR^+^mTregs. As a result, we next examined whether molecules that are known to be involved in TEM are more highly expressed on the surface of the nvTreg after *in vitro* expansion. As shown in Fig. 4B, the expanded nvTregs were found to exhibit both a substantial increase in surface expression of CXCR4, which confers migratory responsiveness to SDF-1, and maintained their high expression of the integrin VLA-4, which interacts with endothelial cell VCAM-1 (Fig. 4C). (All regulatory and effector T cell populations expressed LFA-1, as shown in Supp. Fig. 2A).

We next asked whether the expression of sLe^X^ was also increased on the surface of the *in vitro* expanded nvTregs. However, upon staining for surface expression of sLe^X^, the expanded nvTregs exhibited only a modest increase in expression of sLe^X^ (12% by CD15 s, 22% by HECA452) as compared to freshly isolated nvTregs (Fig. 4D,E). As expression of sLe^X^ can be regulated at the level of reduced installation or enhanced cleavage of fucose on the cell surface, we examined whether the expanded nvTregs exhibited a unique expression profile of the transcripts that encode the enzymes that regulate levels of cell surface α(1,3)-fucosylations. By real-time PCR, we found that the expanded nvTregs did not express mRNA for FUT7 (Fig. 4F) and maintained the high expression of the fucosidase, FUC1, typically found in freshly isolated blood nvTregs (Supp. Fig. 2B). These data indicate that the capacity to express sLe^X^, which is prevalent in memory CD4 T cells populations, is not simply acquired by TCR stimulation in the nvTreg population.

As this extremely low expression of sLe^X^ on expanded nvTregs would result in poor binding to endothelial cells under shear and translate into reduced capacity to suppress tissue inflammation if introduced as a cellular therapeutic, we asked whether enforcing expression of sLe^X^ on the surface of the expanded nvTregs would augment their ability to bind to activated endothelium and thus enhance their potential therapeutic efficacy. If the expanded nvTregs expressed the correct acceptor carbohydrate moieties (sialyl-lactosamine trisaccharides), the addition of fucose in the correct α(1,3)-linkage could produce sLe^X^. To test this notion, viable expanded nvTregs were incubated with a purified, recombinant FTVII enzyme (from R&D Systems/Biotechne), and then stained for sLe^X^ to determine if fucose moieties were added in the correct configuration to the cell surface. The staining results indicated that the FTVII-treated, expanded nvTregs exhibited a dramatically increased expression of sLe^X^ relative to buffer-treated cells (90% from 12%) (Fig. 4G). In addition, not only did the enzymatically treated, expanded nvTregs exhibit increased sLe^X^ expression as indicated by HECA452 staining, but also the cells exhibited a markedly increased capacity to bind E-selectin (when stained under fluid shear conditions, Supp. Figure 2C). As FTVII treatment was able to generate sLe^X^ moieties on the expanded nvTreg, this indicates that the expanded nvTregs express the appropriate scaffold acceptor trisaccharides but lack sLe^X^ due to an absence of α(1,3)-fucose modifications.

The molecules on the expanded nvTregs that were targeted by the FTVII treatment were next examined. FTVII enzyme treatment could generate sLe^X^ determinants on either protein or lipid. However, since a limited pronase treatment of samples of FTVII treated expanded nvTregs effectively removed cell surface sLe^X^, it is apparent that the FTVII treatment generated E-selectin ligands on surface glycoproteins (Supp. Fig. 3A). The identity of the proteins modified by FTVII treatment of expanded nvTregs was determined by performing exhaustive, sequential immuno-precipitation of known sLe^X^ containing proteins from lysates of FTVII treated, expanded nvTregs. The specific immunoprecipitated proteins that expressed sLe^X^ on the expanded nvTregs after FTVII treatment were identified by their capacity to interact with E-selectin by western blot (Fig. 4H). In analyzing all E-selectin binding proteins in the initial lysates, buffer-treated cells showed a single faint E-selectin-reactive band at ~120 kDa, while the FTVII-treated cells exhibited intense E-selectin-binding proteins at ~120 kDa and ~80 kDa. However, after subjecting the total lysate to sequential immuno-precipitation, the FTVII treatment was found to introduce sLe^X^ onto CD44 (~80 kDa), PSGL-1 (a homodimer of ~110–130 kDa monomers) and CD43 (~110–130 kDa) (Fig. 4H), thereby creating the E-selectin ligands known as HCELL, PSGL-1/CLA, and CD4-E, respectively. (Each step in the sequential immunoprecipitation was shown to be complete by western blot analysis of each post stage of immunoprecipitation, see Supp Fig. 4) Importantly, no E-selectin-binding proteins remained in the lysate after successively removing these three proteins.

We next tested whether the enforced generation of E-selectin ligands had functional impact on the ability of the expanded nvTregs to adhere to endothelial cells or to suppress the activation of co-cultured non-regulatory CD4 T cells. As compared to cells treated with buffer only, the FTVII treated, expanded nvTregs exhibited a marked increase in their capacity to roll on and adhere to E-selectin-expressing vascular endothelial cells (Fig. 4I). Importantly, when tested in the typical Treg co-culture assay, which is not dependent on sLe^X^, the FTVII treatment had no effect on the suppressive capacity of the tested Tregs. (Supp. Fig. 3B).

### FTVII-treated nvTregs exhibit enhanced homing to an E-selectin-bearing tissue bed, and enhanced therapeutic activity *in vivo*

**Figure 5:**
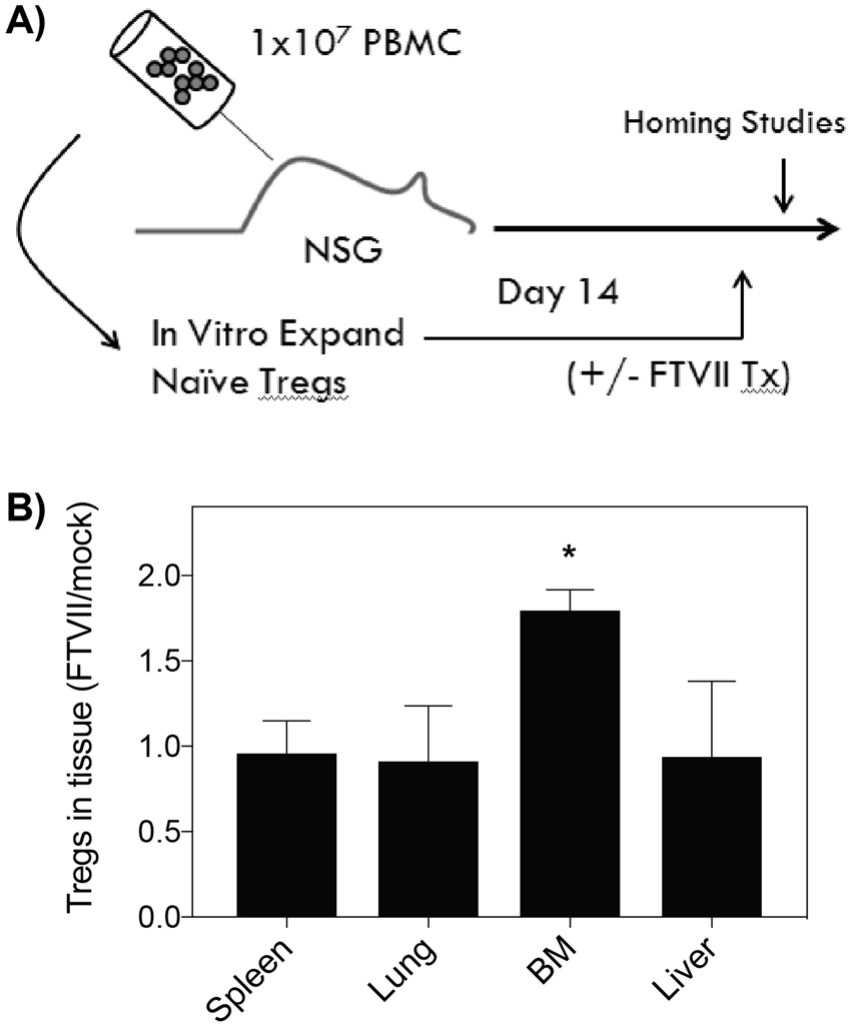
FTVII-Treated regulatory T cells traffic preferentially to bone marrow. (**A**) Depicts the schematic for model of xenogeneic graft vs host disease, noting the simultaneous use of PBMCs for disease initiation and initiation of nvTreg expansion cultures. (**B**) The ability of FTVII treatment to affect tissue migration was tested by co-injecting (i.v.) expanded nvTregs that were buffer or FTVII- treated, and differentially labeled with CFSE or SNARF, into NSG mice 14 days after the injection of 10^7^ PBMCs. After 24 hours, the frequency of Tregs in the bone marrow, lung, spleen, and liver was determined by FACS as compared to the number of human cells in each tissue as determined by staining for human CD45. Data are shown as mean ± SEM. Significance was determined by Student’s t test (*P < 0.05).

Next, we examined whether increasing the expression of surface E-selectin ligands affected the ability of the expanded nvTregs to home to inflamed tissues *in vivo* as would be expected. A xenogeneic model of aGVHD, in which tissue inflammation is initiated in NOD-scid IL2Rγnull (NSG) mice by the injection of human PBMC, was used to determine if buffer-treated or FTVII-treated expanded nvTregs exhibited differential tissue homing ability (see model in Fig. 5A). In using this model, the disease-initiating PBMCs and the potential disease-ameliorating Tregs were derived from the same donor, as a portion of the isolated PBMCs were injected to initiate tissue inflammation in the NSG mice (day 0), and a portion were used to FACS-sort nvTregs that were immediately placed in *in vitro* stimulation cultures. After two weeks of culture, the expanded nvTregs were harvested, tested for purity (FoxP3 stains, Supp. Fig. 3C), treated with buffer alone or with FTVII for exofucosylation, and then injected into mice that had previously received the autologous human PBMCs. To study short-term migration, the PBMC pre-sensitized mice were injected with buffer- or FTVII- treated, expanded nvTregs that had been labeled with CFSE or SNARF before injection. Twenty-four hours after injecting 5 × 10^6^ of the different nvTreg populations, multiple tissues were excised and examined for the presence of the injected human cells, detected via expression of hCD45, SNARF and/or CFSE. As shown in Fig. 5B, the expanded nvTregs that had been treated with FTVII exhibited a significant preferential homing to an E-selectin-expressing tissue (bone marrow) as compared to cells treated with buffer only.

**Figure 6:**
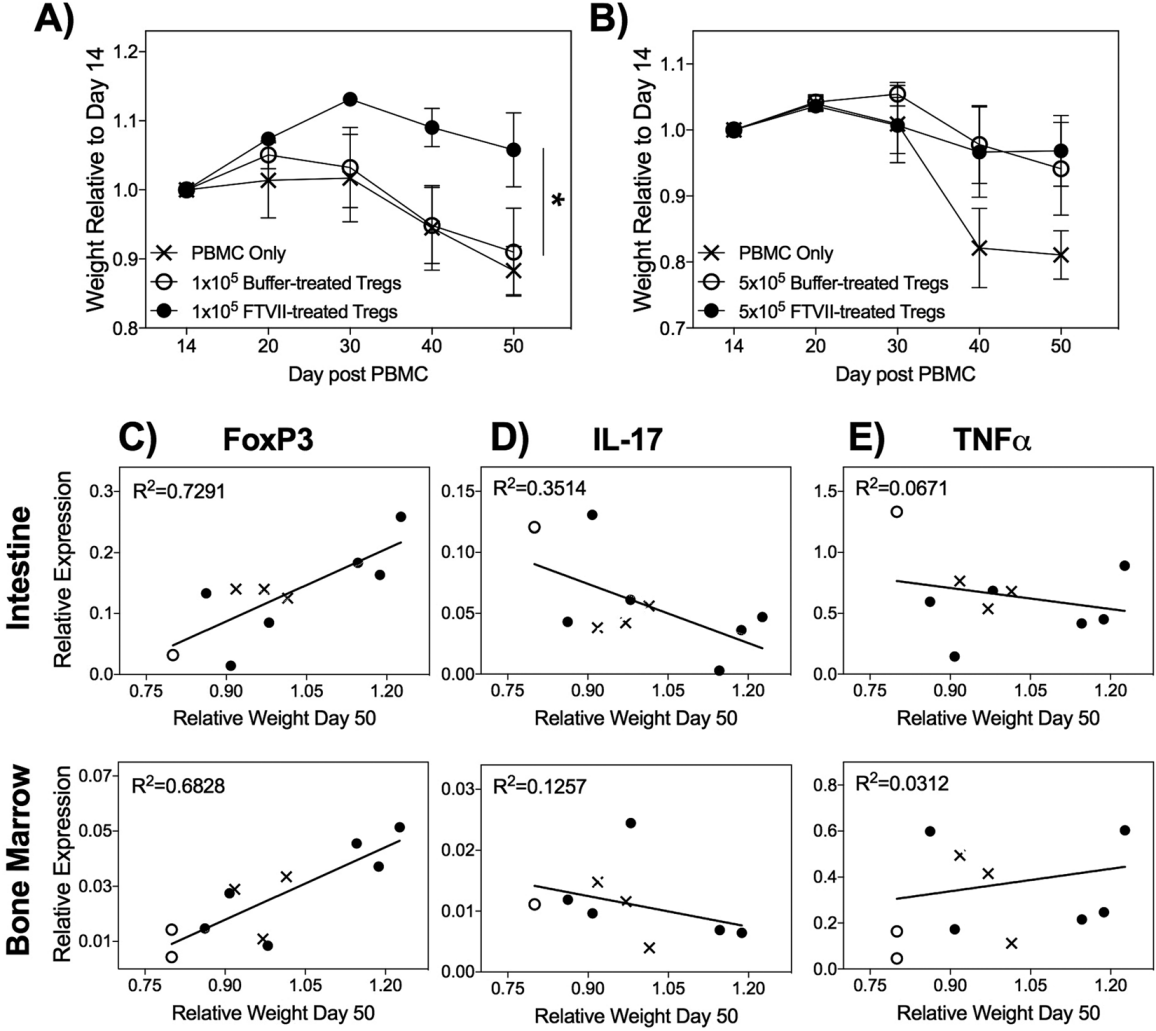
FTVII-treatment improves efficacy of adoptive Treg therapy for GVHD in NSG mice. (**A**) NSG mice that had been injected with PBMC 14d earlier were injected with PBS (PBMC only) or suboptimal numbers (1.0 × 10^5^) of buffer- or FTVII- treated, expanded nvTregs/mouse (n = 6). The injection of FTVII-treated/expanded nvTregs significantly reduced the weight loss that is indicative of xenogeneic GVHD severity in NSG mice^46^. (**B**) In contrast, when mice were given optimal (5.0 × 10^5^) doses of buffer- or FTVII- treated nvTregs, all Treg injected mice showed similar rescue from disease. RNA, isolated from the intestine (top) and bone marrow (bottom) isolated from mice that were still viable on day 50 after receiving the indicated treatments was examined for expression of (**C**) FoxP3, (**D**) IL-17, and (**E**) TNF-α relative to B_2_M by real-time PCR. Correlation to weight loss as a measure of disease activity was determined by linear regression analysis. Data shown are mean ± SEM. Significance, determined by Student’s t test, is indicated by (*P < 0.05), (**P < 0.01), and (***P < 0.001).

To determine whether administering nvTregs that had been exofucosylated would result in enhanced therapeutic activity, suboptimal numbers of expanded nvTregs were tested for their ability to inhibit PBMC-mediated xenogeneic GVHD. After administering a low number (1 × 10^5^) of buffer-treated or FTVII-treated expanded nvTregs into mice that had previously received the autologous PBMCs, survival and weight loss were measured as indicators of aGVHD severity^46^. Under these conditions, mice injected with exofucosylated nvTregs exhibited a significant reduction in disease severity as compared to mice that received the buffer-treated nvTregs or PBS (Fig. 6A). In contrast, by injecting optimal numbers (5 × 10^5^) of mock-treated or FTVII-treated expanded nvTregs, the advantage conferred by the previous administration of exofucosylated Tregs was no longer detectable (Fig. 6B). Thus, the nvTregs treated with FTVII only exhibited their beneficial ability to inhibit disease when injected at suboptimal numbers. This suggests that the FTVII treatment conferred a potent *in vivo* amplification of Treg function – a finding that has direct implications for the problem of limiting cell numbers of Treg cellular therapies.

In addition to relating the administration of Tregs to disease activity as indicated by weight loss and prolonged survival^46^, we determined if the administration of the Treg therapeutic could also be shown to affect the expression of human pro-inflammatory cytokines by the disease initiating PBMCs. To perform these studies, RNA isolated from various aGVHD relevant tissues (taken on day 50) was interrogated for expression of human cytokine genes by RT-PCR. (However, as this analysis could only be performed on mice that lived to day 50 post-transplant, there were limited numbers of samples from the mice exhibiting acute disease that had received only PBMCs and PBS.) The samples were examined for expression of human FoxP3, as well as the human pro-inflammatory cytokines IL-17 and TNFα, to determine if there was a relationship between disease activity (as indicated by body weight) and expression of these genes at this late time point. As shown in Fig. 6C, increased expression of human FoxP3 was detected in the intestine and bone marrow in the healthier, Treg-recipient mice as compared to the mice exhibiting weight loss. In contrast, the analysis of human IL-17 expression revealed the opposite result in which IL-17 expression was directly associated with disease activity (Fig. 6D). Lastly, there was no association between expression of TNFα (Fig. 6E) and GVHD severity.

## Discussion

The ability to exploit Treg-based therapy for immunological diseases offers the exciting opportunity to establish immune homeostasis and/or enable tolerance without the attendant risks of infection and organ toxicities inherent to pharmacologic immunosuppressants. Here we report the study and the functional comparison of specific human PBMC-derived Treg subpopulations that had been expanded *in vitro*. Our investigations focused on several critical aspects of human Treg biology with intent to determine if any one subset might exhibit advantages in purity, lineage fidelity, migration capacility, and suppressive activity; features that would be requisite for optimizing Treg therapeutics. In addition to offering information relevant for Treg therapeutic development, these data also shed light on fundamental aspects of Treg glycome biology. Specifically, although stimulated nvTregs mirror memTregs in suppressive ability, we observed that this stimulation does not induce the naïve Tregs to become bonafide memory Tregs. Instead, by maintaining their regulation of fucose-regulating enzymes, the *in vitro* expanded nvTregs retain their naïve expression of surface sLe^X^ glycosylation. How the expression of fucose modifying enzymes is regulated when nvTregs transition into memory Tregs *in vivo*, is as yet unclear.

Over the years, human Tregs have been divided into many different subsets defined by the expression of various surface proteins and usually associated with distinct functional attributes. The most basic subdivision of human Tregs separates the CD4 + CD25^hi^CD127^10^ Tregs into memory or naïve populations by virtue of the expression of CD45RA (naïve) or CD45RO (memory). By studying the gene expression profiles in over 40 reported Treg subset divisions, a recent study concluded that human naïve Tregs and DR+ memory Tregs truly represented distinct subsets^47^. As our primary goal in these studies was to determine whether distinct Treg subsets exhibited differential suitability for use as a cellular therapeutic, we tested the functional properties of human naïve, DR+ and DR− memory Treg subpopulations both after fresh isolation from blood and after *in vitro* expansion.

One major consideration was that Treg-based therapeutics would require a large number of cells, and thus the ideal Treg source had to be highly proliferative. The total population of memory Tregs is known to include most of the Tregs that express sLe^X^ and those that also exhibit the capacity to transition into pro-inflammatory cells that co-express IL-17 or IFNγ^4,36^. Here, we report that unlike the DR^−^mTregs, the vast majority of effector DR + mTregs express sLe^X^ and exhibit strong transendothelial migration (TEM) ability, indicating that the most suppressive effector Tregs (DR^+^mTreg) are also the cells that are the most prone to enter tissues. Unfortunately, the data also indicate that the highly suppressive DR^+^mTregs are not a suitable therapeutic choice as, although these circulating “effector” Tregs exhibit the highest expression of sLe^X^, the strongest suppressive capacity, and the greatest ability to transmigrate across endothelium under remarkably high shear forces, they only constitute a small fraction of blood-resident Tregs and cannot be sufficiently or stably expanded *in vitro*. The fact that these cells are unable to proliferate may be a beneficial property *in vivo*, in that permitting unrestricted proliferation of highly suppressive Tregs in tissues could have deleterious effect(s) on the host.

In contrast, although the DR^−^mTregs were more proliferative than their DR+ counterparts, they too were not an ideal choice as they gave rise to cells that co-expressed pro-inflammatory cytokines and FoxP3. In contrast, the nvTregs were only weakly suppressive when tested *ex vivo*, but exhibited the greatest proliferative capacity, maintained the highest lineage fidelity with low pro-inflammatory plasticity (i.e., IL-17 production) and became markedly more suppressive after *in vitro* expansion. Yet, both the freshly-isolated and culture-expanded nvTregs were found to lack expression of α(1,3)-fucosyltransferases necessary for creation of sLe^X^ motifs that mediate initial cell attachment to endothelium under hemodynamic shear. However, having found that the nvTregs express proteins decorated with sialyllactosamines, the acceptor trisaccharide which is modified by α(1,3)-linked fucose (by action of α(1,3)-fucosyltransferases) to produce sLe^X^, we reasoned that sLe^X^ could be enforced on the nvTreg surface by exofucosylation using FTVII and donor nucleotide sugar (GDP-fucose). Treating the expanded sLe^X^ negative nvTregs with the fucosyltransferase FTVII resulted in their expression of sLe^X^, had no adverse affects on their *in vitro* suppressive ability, and strongly enhanced their *in vivo* inhibition of aGVHD in the PBMC/NSG xenogeneic model. These results not only indicate that the expanded nvTreg are highly efficient *in vivo*, but also that if they were fucosylated, markedly less sLe^X+^ (fucosylated) nvTregs were needed to inhibit aGVHD compared to the activity of the unfucosylated nvTregs. This finding suggests that reduced numbers of Tregs could be efficacious as a cellular therapy, if they are first α(1,3)-exofucosylated treated to enhance surface sLe^X^ expression.

As current Treg trials indicate the need for improvement, strategies to enhance Treg function/fidelity may help to realize the promise of adoptive Treg therapy^12^. Our data show that by selecting a Treg population from adult PBMCs to be the source of therapeutic Treg development, it may be possible to generate autologous Treg therapeutics with the potential for long-term engraftment. A number of recent studies have reported attempts to utilize Tregs derived from 3^rd^ party cord blood to clinically inhibit the effects of immune-mediated/inflammatory diseases^48,49^, including a study inducing selectin ligands on the surface of these cells^50^. Yet, cord blood-derived Tregs are less suppressive than those from adult blood^51^. Furthermore, although Treg suppression is MHC-independent^52^, if the goal of the therapeutic is to induce long-term Treg engraftment, T-cell/Treg engraftment has been shown to require autologous MHC in the antigen presenting cell compartment^53^. This would indicate that cellular therapeutics derived from allogeneic third party PBMC- or cord blood- derived Tregs may only satisfy short-term requirements for Treg suppression *in vivo*. In contrast, Treg engraftment may be more robust and functionally potent if the Treg cellular therapeutic was either derived from the expansion of patient-derived autologous Tregs, or, in cases of allogeneic bone marrow transplantation (HSCT), Tregs propagated from PBMCs derived from the hematopoietic stem cell donor. Accordingly, we focused here on examining the therapeutic suitability of adult PBMC-derived Treg subsets to increase the feasibility of providing autologous Treg cellular therapies to patients suffering from autoimmune diseases or to recipients of HSCT suffering from aGHVD.

Collectively, our findings highlight the use of culture-expanded adult PBMC-derived naïve Tregs in combination with cell surface glycan engineering to enforce sLe^X^ expression to direct the administered Tregs to sites where they are needed. Surprisingly, our data also suggests that memory Tregs should be purposefully excluded from the source used to derive the Treg therapeutic, as the memory Treg subsets exhibit increased propensity to become ex-Tregs or pro-inflammatory cells. Thus, in addition to adding to our knowledge of human Treg immunobiology and human Treg glycobiology, the data provide a readily translatable roadmap for optimizing Treg-based therapeutics.

## Methods

### PBMC and Human Treg isolation

Peripheral blood was drawn from healthy individuals after informed consent, with approval by the Institutional Review Board at the Brigham and Women’s Hospital. All experiments were performed in accordance with the relevant guidelines and regulations in these protocols. Peripheral Blood Mononuclear Cells (PBMCs) were isolated with Ficoll Hypaque (GE Healthcare) gradient centrifugation. CD4^+^ T cells, isolated by human CD4 T cell Isolation kit (negative selection, Miltenyi Biotec), were stained with antibodies to CD25 (clone M-A251), CD127 (clone HIL-7R-M21), CD45RA (clone HI100) and HLA-DR (clone G46–6) from BD Biosciences, and sorted on a FACSAria (BD Biosciences). nvTreg (CD45RA^+^), and memory (CD45RA^−^) Tregs are isolated as CD25^HI^ and CD127^LO^, while the HLA-DR^−^ and HLA-DR^+^ Treg differ by HLA-Class II expression. Memory Tresp (non –regulatory memory CD4 T-cells) are isolated as CD25^LO^, CD127^HI^, and CD45RA^−^.

### *In vitro* Treg expansion cultures

Freshly isolated, blood-derived T cell populations were cultured in 96-well round bottom plates (Costar) coated with 0.5 ug/ml anti-CD3 (UCHT-1, eBioscience) with irradiated antigen presenting cells (iAPC, PBMCs depleted of T cells via anti-CD2-beads from Dynal/Invitrogen) and 0.25 ug/ml anti-CD28 (clone 28.2 eBioscience) in RPMI-1640 medium (Life Technologies) supplemented with 10% FBS (Life Technologies) and 20 U/ml rhIL-2. Confluent wells were split into new plates and IL-2 containing media was supplemented every 2–3 days. After two weeks, purity (expression of FoxP3^high^) was tested by flow cytometry using anti-FoxP3 (clone 206D, BioLegend) and the intracellular FoxP3 staining kit (Biolegend).

### Co-culture suppression assays

Freshly isolated Tresp cells (2.5 × 10^3^/well, CFSE labeled [Invitrogen]) were co-cultured with 1.25 × 10^3^/well Treg, iAPCs (1 × 10^4^/well) at a 1:1/2 ratio, in triplicates in wells coated with 0.2μg anti-CD3 (UCHT-1, eBioscience), or in wells given beads coupled with anti-CD3 (UCHT1, BD BioSciences) and anti-CD2 (RPA-2, BioSciences) or anti-CD28 (28.2, BioSciences) (tosyl-activated Beads from Dynal. Suppressive capacity was determined after 4 or 5 days by analyzing CFSE dilution on a BD Canto II with proliferation index analysis using FlowJo (Treestar).

### Antibodies, flow cytometric analysis and immunoprecipitation

Flow cytometry was performed on a FACS Canto II (BD Biosciences) and analyzed using FlowJo Software (Treestar). Cells were stained with antibodies to CD3, CD4 (OKT4), CXCR4 (12G5), VLA-4 (9F10) and CD15s (CSLEX1) from BD biosciences, and HECA452, CD45 (2D1), CD44 (BJ14), CD162 (KPL-1), CD43 (CD43-10G7), and FoxP3 (206D) from Biolegend. Staining with mouse E-selectin Ig chimera (RnD Systems) was detected with either anti-His FITC (Bethyl Laboratories) for flow cytometry or secondary rat anti-mouse CD62E (BBIG-E4, RnD Systems) followed by goat anti-rat IgG HRP (Southern Biotech) for western analyses.

Lysates were made from cells by sonication/vortexing in buffer containing 50 mM Tris, 150 mM NaCl, 20 ug/ml phenylmethanesulfonyl fluoride (PMSF), 0.2% NaN_3_, protease inhibitor cocktail (Roche), 2% NP40, and 0.2% SDS. Precleared lysates were incubated antibodies to CD43 (1G10, L60, BD Biosciences; 20819, Santa Cruz Biotechnology), PSGL-1 (KPL-1, BD Biosciences; 20929, Santa Cruz Biotechnology), or CD44 (515, BD Biosciences; 2C5, RnD systems). Protein was immunoprecipitated with Protein-G agarose (Life Technologies). Western Blots were run with Reducing SDS-PAGE gels (Bio-Rad).

### Exofucosylation

Cells were treated with fucosyltransferase VII (FTVII) or buffer-only at 37 °C for 60 minutes. Tregs were resuspended at 3 × 10^6^ cells/30 ul in HBSS with 2 ug FTVII (RnD Systems), 10 mM Hepes, 0.1% Human Serum Albumin, and 1 mM GDP fucose (Carbosynth). Treatment success was determined by flow cytometry with HECA452 mAb (Biolegend).

### Parallel Plate Flow Chamber

Human umbilical vein endothelial cells (HUVEC, Lonza) were cultured in EGM-2 media (Lonza) in Bioflux microfluidic chambers coated with 250 ug/ml fibronectin (BD Biosciences). Four hours prior to rolling assay, cells were activated with 40 ng/ml TNFα (RnD Systems). Different cell subsets were infused into the chamber in a bolus of 10 × 10^6^/ml – shear stress was applied from 0.5–8 dynes/cm^2^ and the number of rolling/adherent events relative to counted cells in initial field of view was counted (to normalize for rolling capacity on endothelium).

### GVHD model and homing assays

NOD/SCID IL-2Rγnull mice (NSG, Jackson Laboratories, 4–8 weeks of age) were given intraperitoneal (IP) PBMCs (10 × 10^6^) from healthy donors. 14 days later, mice were given expanded nvTregs that had been either buffer- or FTVII-treated by intravenous (IV) injection. The mice were monitored until day 50 for signs of GVHD – weight loss, hunched posture, hair loss, etc. At day 50, or at >20% weight loss, mice were sacrificed and bone marrow and intestine were harvested for analysis by flow cytometry and PCR. Mice were not included in the analysis if they failed engraftment of the injected PBMCs as indicated by fewer than 1 × 10^4^ human cells in the bone marrow, spleen or lung on d50. The homing assays were performed on NSG mice given PBMCs 14 days prior to injection of the labeled Treg populations. The mice were subsequently injected (i.v.) with buffer-treated and FTVII-treated naïve expanded regulatory T cells that were differentially stained with either CFSE or SNARF (Life Technologies). (The staining dyes were alternated in repeat experiments as a control). At 24 hours, mice were sacrificed and single cell suspensions of spleen, bone marrow, lung and liver were analyzed for frequency of CFSE^+^ or SNARF^+^ cells within the population of cells expressing hCD45 (Biolegend). All animal methods and experimentations were performed in accordance to our accepted protocols following the guidelines outlined by the Harvard Medical School Center for Comparative Medicine Institutional Animal Care and Use Committee-approved protocols.

### Transmigration

HUVEC cells (Lonza) were cultured in EBM-2 media with EGM-2 supplements (Lonza) in 5.0 micron transwells (Costar) in 24 well plates overnight. Transwells were pretreated with 100 ug/ml Fibronectin (BD Biosciences) for 2 hours at 37 °C. HUVECs were activated for 4 hours with 40 ng/ml TNFα (RnD Systems) and placed in wells with lower well containing 125 ng/ml SDF-1. Tregs were overlayed onto the HUVEC layer and incubated at 37 °C for 12 hours. Cells in the lower chamber were counted by flow cytometry and normalized with counting beads to cell input (based on cell number in adjacent, transwell-free lane) – subtracting the number of cells migrating through unactivated HUVEC.

### PCR analysis

mRNA was isolated using RNeasy Microkit (Qiagen) from 10% of each mouse organ and ~100 ul whole blood. cDNA was generated using VILO cDNA Synthesis Kit (Life Technologies). Realtime PCR was performed using the Taqman fast PCR master mix (Life Technologies), with primers for Beta-2 Microglobulin, IL-17, TNFα, FoxP3 and IFNγ (Life Technologies). Fucosyltransferase and fucosidase experiments were run with oligos to FUT4 (Origene), FUT7^54^, and FUC1α^55^, with SYBR Select Master Mix (Life Technologies). Oligonucleotide sequences for the RT-PCR detection of FUT4, FUT7, and FUC1 are shown in Supp Table 1. All experiments performed on a Lightcycler 480 (Roche) and analyzed using StepOne software (Life Technologies).

### Pronase Treatment

Cells were treated with 1 mg/ml Pronase (Roche) in HEPES Buffer at 37 °C for 30 min.

## Electronic supplementary material

Supplementary Information
