# Supplementary material for: Optimizing human Treg immunotherapy by Treg subset selection and E-selectin ligand expression
Source: Sci Rep. 2018 Jan 11;8:420. doi: 10.1038/s41598-017-17981-z (PMC5765004; doi:10.1038/s41598-017-17981-z)
Supplement: Supplementary file 1 — Supplementary Information [file 41598_2017_17981_MOESM1_ESM.pdf]

Optimizing human Treg immunotherapy by Treg subset selection and E-selectin ligand expression

Conor Donnelly[2,4,5], Brad Dykstra[2,4, 6], Nandini Mondal[2,4], Junning Huang[1], Belinda J. Kaskow[1], Russell Griffin[2], Robert Sackstein[2,3,4], and Clare Baecher-Allan[1, 2]

[1]Ann Romney Center for Neurologic Disease, Harvard Medical School, Boston, MA 02115;
[2]Department of Dermatology, and [3]Department of Medicine, Brigham and Women's Hospital, Boston, Harvard Medical School, MA 02115; [4]Program of Excellence in Glycosciences, Harvard Medical School, Boston, MA 02115, [5]University of Pittsburgh School of Medicine, Pittsburgh PA; [6]Platelet Biogenesis, Boston, MA;

SUPPLEMENTAL DATA

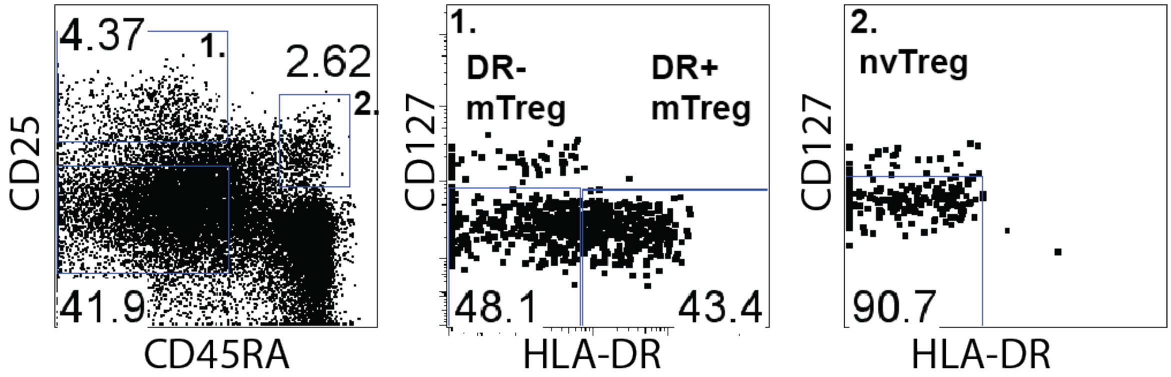

Supplemental Figure 1: Treg and Tresp sorting strategy. Total CD4 T cells were first isolated from PBMCs via negative selection by Miltenyi Biotech CD4 negative isolation kit II, then FACS sorted. Naïve Tregs were CD25^{hi} CD45RA^{+}CD127^{lo}HLA-DR^{-}; DR-mTregs were CD25^{hi} CD45RA^{-}CD127^{lo}HLA-DR^{-}; DR+mTregs were CD25^{hi} CD45RA^{-} CD127^{lo}HLA-DR^{+}; mTresp were CD25^{lo} CD45RA^{-}CD127^{hi}.

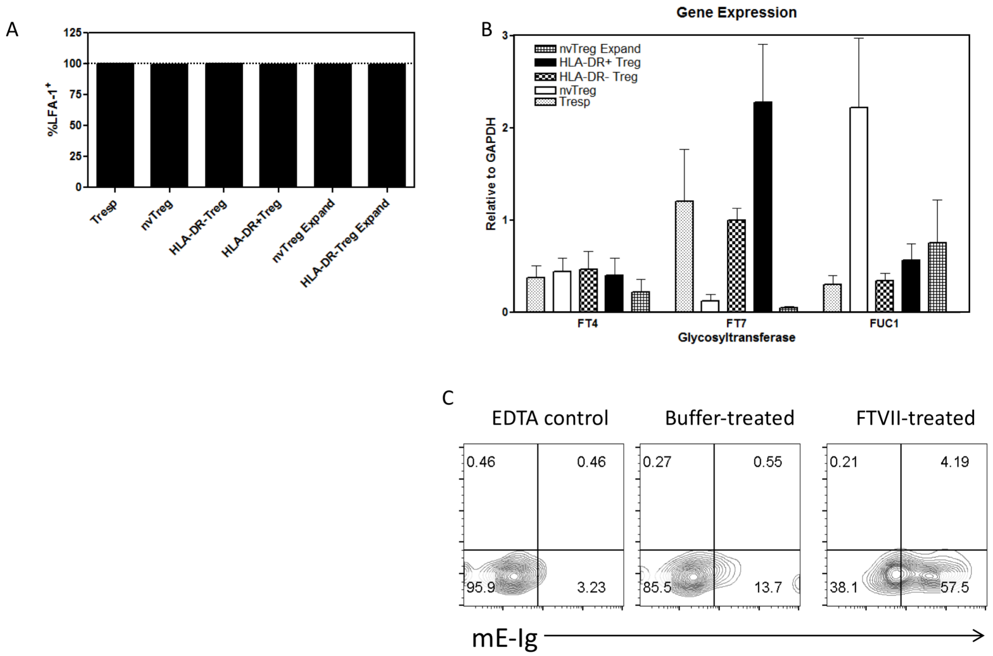

Supplemental Figure 2: (A) LFA-1 staining of "freshly isolated" and expanded Treg subsets. (B) The ex vivo freshly isolated T cells subsets and 14-day in vitro expanded nvTregs were tested for their levels of expression of FT-IV, FT-VII, and FUC1. (C) Expanded nvTreg, buffer-treated or FTVII-treated, were stained with mouse E-selectin-Ig with EDTA control on the far left (binding is calcium dependent) to demonstrate the induced expression of cell surface sLe^x was functional.

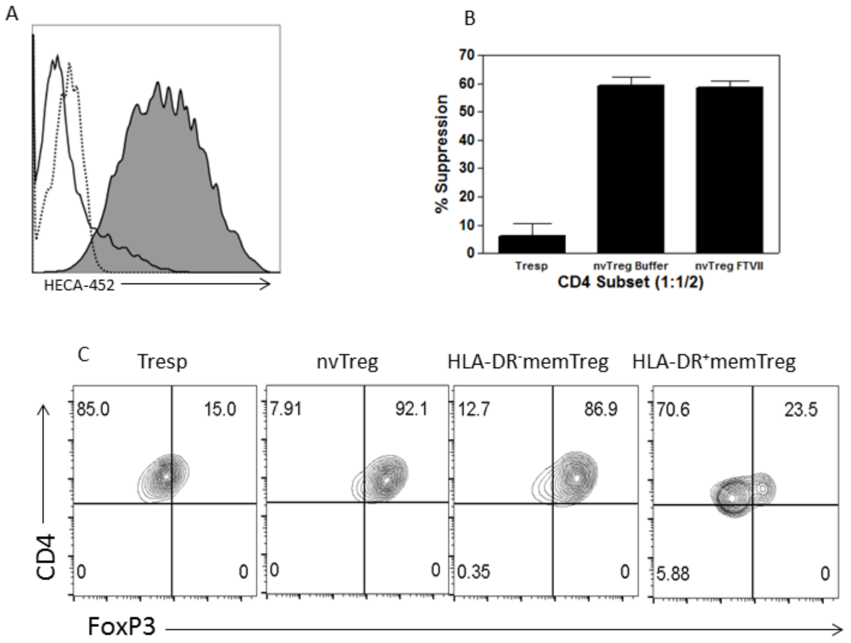

Supplemental Figure 3: (A) FTVII-treated cells (gray histogram) were treated with pronase (white histogram) and stained with HECA-452 mAb demonstrating that FTVII treatment primarily induced sLex moieties on cell surface proteins. (B) Buffer- or FTVII-treated expanded nvTregs showed equivalent ability to suppress Tresp proliferation. (C) Plots showing the typical FoxP3 staining on the various CD4+ subsets after two weeks of in vitro expansion.

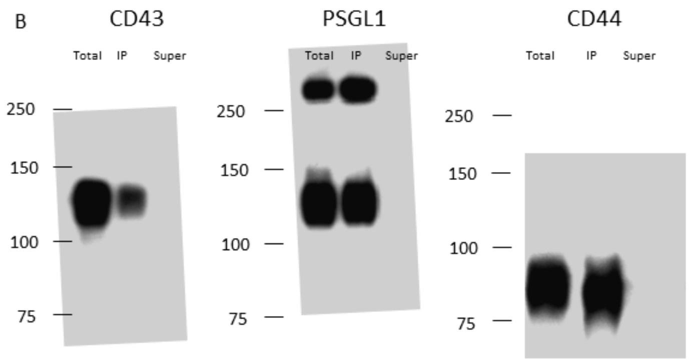

Supplemental Figure 4. Each step in the sequential immunoprecipitation studies was tested and shown to have been a complete immunodepletion of the indicated proteins as each could no longer be detected in the supernatant that was then immunoprecipitated with the subsequent mAbs until no e-selectin ligand binding proteins remained in the lysates as shown in Figure 4G in the accompanying manuscript. The mAbs used in precipitating and detecting the different sLe^x scaffold proteins were: CD43 (1G10, L60, BD Biosciences; 20819, Santa Cruz Biotechnology), PSGL-1 (KPL-1, BD Biosciences; 20929, Santa Cruz Biotechnology), and CD44 (515, BD Biosciences; 2C5, RnD systems).
(Note these sections of western blots were cropped from the whole blot films shown below, the lanes were not altered in any manner)

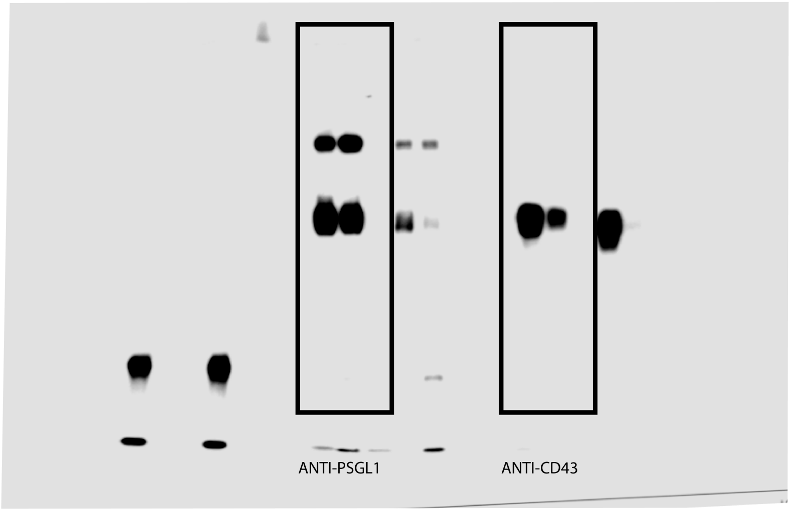
ANTI-PSGL1
ANTI-CD43

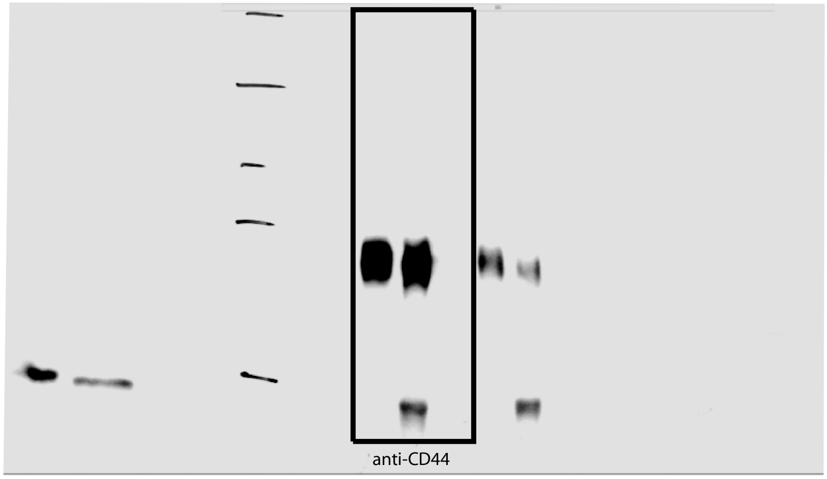
anti-CD44

Supplemental Table 1

Protein	Forward Primer (5’ –3’)	Reverse Primer (5’ –3’)
FTIV	GGGTTTGGATGAACTTCGAGTCG	GGTAGCCATAAGGCACAAAGACG
FTVII	TCCGCGTGCGACTGTTC	GTGTGGGTAGCGGTCACAGA
Fucosidase1	AGTCACCCTGTTGCCTATGG	TTTGGCGCTTTTAGATTGCT

Supplemental Table 1: Oligos used for detection of fucosyltransferase 4 (FUT4), fucosyltransferase 7 (FUT7) and fucosidase 1α, (FUC1).
